# Stability of uniformly labeled (^13^C and ^15^N) cytochrome *c* and its L94G mutant

**DOI:** 10.1038/s41598-021-86332-w

**Published:** 2021-03-24

**Authors:** Abdullah Naiyer, Bushra Khan, Afzal Hussain, Asimul Islam, Mohamed F. Alajmi, Md. Imtaiyaz Hassan, Monica Sundd, Faizan Ahmad

**Affiliations:** 1 Centre for Interdisciplinary Research in Basic Sciences, Jamia Millia Islamia, Jamia Nagar, New Delhi, 110025 India; 2 Department of Pharmacognosy College of Pharmacy, King Saud University, Riyadh, 11451 Kingdom of Saudi Arabia; 3 NMR-II Lab, National Institute of Immunology, Aruna Asaf Ali Marg, New Delhi, 110067 India

**Keywords:** Biological fluorescence, Biophysical chemistry, Protein folding

## Abstract

Cytochrome *c* (cyt *c*) is widely used as a model protein to study (i) folding and stability aspects of the protein folding problem and (ii) structure–function relationship from the evolutionary point of view. Databases of cyts *c* now contain 285 cyt *c* sequences from different organisms. A sequence alignment of all these proteins with respect to horse cyt *c* led to several important conclusions. One of them is that Leu94 is always conserved in all 30 mammalian cyts *c*. It is known that mutation L94G of the wild type (WT) horse cyt *c* is destabilizing and mutant exists as molten globule under the native condition (buffer pH 6 and 25 °C). We have expressed and purified uniformly labeled (^13^C and ^15^N) and unlabeled WT horse cyt *c* and its L94G mutant. We report that labeling does not affect the thermodynamic stability of proteins. To support this conclusion, the secondary and tertiary structure of each protein in labeled and unlabeled forms was determined by conventional techniques (UV–Vis absorption and circular dichroism spectroscopy).

## Introduction

Folding of a protein from its structureless state to its unique native 3D state on the physiological time scale occurs via a number of intermediates^1–3^. Two of these folding intermediates, namely molten globule (MG) and pre-molten globule (PMG) are well characterized^4^. These folding intermediates can be trapped in the presence of low concentrations of chemical denaturants, and at extreme pH and temperature. Some other procedures such as chemical modification, site-directed mutagenesis (or point mutation) and cleavage of the covalent bond of natural proteins also yield folding intermediates in dilute buffer (near neutral pH) at 25 °C. These observations suggest that there are at least four thermodynamic states on the protein folding pathway, i.e., N (native, folded) ↔ MG ↔ PMG ↔ D (denatured, unfolded). Based on results from theories, simulations and wet-lab experiments, it became evident that MG state has the following common structural characteristics^4–6^. (i) The presence of a substantial amount of stable secondary structure (85–100% of the native secondary structure), as revealed by the far-UV circular dichroism (CD). (ii) Full or partial loss of tertiary interactions produced by tight packing of side chains, as revealed by the near-UV CD. (iii) A largely globular state having a radius of gyration which is only 10–30% more than that of the native state as revealed by hydrodynamic measurements such as dynamic light scattering, viscosity and size exclusion chromatography. (iv) The presence of substantial amount of newly exposed hydrophobic patches which can bind to hydrophobic dyes. Cytochrome *c* (cyt *c*) has been used as a model protein to study the effect of denaturants and mutations on the structure and stability of proteins^7–13^. We have previously shown that L94G mutant of horse cyt *c* displays common structural characteristics of molten globule (MG) state in the native buffer at pH 6.0 and 25 °C^14^.

It is known that hydrogen bond in labeled water (D_2_O) is stronger than that in normal (unlabeled) water^15^. To know whether labeling of proteins atoms affects protein stability, we have expressed and purified uniformly labeled (^13^C and ^15^N) and unlabeled wild type (WT) horse cyt *c* and its L94G mutant. We have measured the thermodynamic stability of each protein in terms of both *T*_m_ (midpoint of denaturation) and Gibbs free energy change (*∆G*_D_^0^ at 25 °C). It has been observed that labeling of either WT protein or its mutant does not affect its thermodynamic stability. To interpret these observations, we have determined the structure of unlabeled and labeled forms of both proteins using conventional techniques (UV–Visible absorption spectroscopy, circular dichroism and dynamic laser scattering).

## Materials and methods

### Site-directed mutagenesis, cloning and protein expression

The *Escherichia coli* strain DH5α was used for cloning and amplification of the DNA whereas *Escherichia coli* BL21 (DE3) competent cells were used as host to express unlabeled (^14^N,^12^C) and labeled (^15^N,^13^C) WT horse cyt *c* and its mutant L94G as described previously^16^. The expression plasmid pBTR encodes for cyt *c*, and cyt *c* heme lyase (CCHL) was a kind gift from Professor Gary Pielak, University of North Carolina, USA. The expression plasmid, pBTR(hCc) is a derivative of the pBTR1 expression vector containing constitutive or inducible promoter directing the expression of protein^17^. Heme lyase is essential for the covalent attachment of the heme to cyt *c* polypeptide chain. An upstream *lac* promoter controls the expression of both cyt *c* and CCHL, and a gene coding *β*-lactamase for ampicillin resistance (Amp^r^) is used for bacterial colony selection. L94G mutation was introduced by PCR-based Quick-change site-directed mutagenesis (Stratagene, USA), using the plasmid pBTR as a template.

### Purification of unlabeled (^14^N ^12^C) WT horse cyt c and its L94G mutant

The WT cyt *c* and its L94G mutant were purified according to the protocol given by Patel et al.^17^ with a minor modification^18^. Briefly, inoculation (1%) of 1 L of nutrient-rich medium (12 g bactotryptone, 24 g yeast extract, 5 ml glycerol, 2.3 g KH_2_PO_4_ and 12.5 g K_2_HPO_4_) was done with overnight primary culture. Ampicillin was added to the culture to a final concentration of 100 mg/L. Cells were grown overnight with continuous shaking at 200 rpm for 30 h and 37 °C. Cells were pelleted down at 4000 rpm for 30 min and 4 °C. The cell pellet (3 ml/g cell paste) was resuspended in lysis buffer (pH 6.8) containing 50 mM tris (hydroxymethyl) aminomethane–Cl and 1 mM EDTA. Cells were sonicated on ice (Nugen Scientific Sonicator) with 60 pulses of 10 s at an interval of 30 s. The lysate was then centrifuged at 8000 rpm for 40 min at 4 °C and the supernatant was collected. Ammonium sulphate was added to the supernatant to a final concentration of 300 g/l while stirring the mixture on ice slowly, and the mixture was centrifuged at 8000 rpm for 40 min at 4 °C. Finally, the supernatant was collected and dialyzed against distilled water at 4 °C with two to three changes every 12 h. The dialysate was concentrated on a 3-kDa amiconfilter (Millipore, USA) to less than 10 ml and dialyzed against low-salt buffer (0.878 g/l NaH_2_PO_4_, 3.656 g/l Na_2_HPO_4_) on the same 3-kDa amiconfilter. The dialysate was loaded onto a pre-equilibrated Resource S (cation exchange column). The wild type and mutant proteins were eluted with high-salt buffer (0.652 g/l NaH_2_PO_4_, 4.096 g/l Na_2_HPO_4_, 58.44 g/l NaCl) using a linear gradient. Fractions of 3 ml each were collected. All fractions having an absorbance (A) ratio (A_410_/A_280_) greater than 4 were pooled in one tube. The sodium dodecyl sulfate polyacrylamide gel electrophoresis (SDS-PAGE) was performed to check the purity of proteins.

### Purification of ^15^N^13^C labeled WT horse cyt c and its mutant L94G

Purification of ^15^N ^13^C labeled WT horse cyt *c* and its mutant L94G were conducted in *E. coli* as described previously^16^. Cells were harvested and sonicated, followed by ion-exchange chromatography (SP-sepharose/Mono-S) and gel filtration chromatography. The SDS-PAGE was performed to check the purity of proteins.

### Gel electrophoresis

The purity of the proteins was assessed by SDS-PAGE using the procedure developed by Laemmli^19^. 14% resolving gels in 375 mM Tris–HCl buffer (pH 8.8) containing 4% stacking gel in 125 mM Tris–HCl buffer (pH 6.8) were run in SDS-PAGE running buffer (pH 8.8) at a constant current of 30 mA. Proteins were visualized by staining gels with *Coomassie Brilliant Blue R*-*250*.

### Preparation of dialysis tubing

Dialysis tubing with molecular mass cut-off (6–8 kDa) was purchased from Sigma Chemical Co. (St. Louis, USA). Pieces of dialysis tubing (4–5 inches) were prepared according to the procedure given by Dr. P. M^c^Phie^20^. First, pieces of the tubing were soaked in 50% ethanol for one hour and then thoroughly washed and immersed in NaHCO_3_ and EDTA solution (mM ratio of 10:1) for 30–40 min at 60 °C. To prevent attack by cellulolytic microorganisms, pieces of the tubing were then washed thoroughly with distilled water and stored in 70% alcohol at 4 °C. These treatments remove all impurities such as glycerin, trace of sulfurous compounds and heavy metal ions from the cellophane tubes^20^. Each piece of the tubing was thoroughly washed with distilled water before filling protein solution in it.

### Preparation of the native buffer

Protein samples for measurements in the native buffer (30 mM sodium cacodylate buffer (pH 6.0) containing 100 Mm NaCl) were prepared. The pH of the buffer solution was adjusted by adding small amounts of HCl or NaOH as per requirement using pH meter (Orion 2 star) supplied by Thermo Scientific Industries Pvt. Ltd., India. The buffer was filtered through the Whatman filter paper No. 1 and stored at 4 °C for further use.

### Preparation of protein stock protein solution

Cyt *c* exists in either oxidized form (Fe^3+^) or reduced form (Fe^2+^). The reduced form has a tendency to auto-oxidize after denaturation, thus, making the process irreversible^21^. This is the reason why the oxidized form is best suited for equilibrium denaturation studies. The purified proteins were oxidized by adding 0.1% K_3_Fe(CN)_6_ (potassium ferricyanide) as mentioned earlier^22^. Finally, to remove all the excess of K_3_Fe(CN)_6_, the solution of the protein was dialyzed against several changes of 0.1 M NaCl solutions at 4 °C and pH 7. The extent of oxidation was confirmed by the disappearance of two sharp α and β-peaks at 550 and 520 nm that are signature peaks of the reduced cyt *c*^23^ and the appearance of a new peak at 530 nm in the absorption spectrum of the protein. The oxidized protein stock solution was then filtered through a Millipore filter of pore size 0.22 μm, and this stock solution was always stored at 4 °C for further use. The concentration of WT cyt *c* and its mutant L94G was determined using a value of 106,100 M^−1^ cm^−1^ for the molar absorption coefficient (*ε*) at 410 nm^24^. Samples of each protein were prepared in 30 mM cacodylate-100 mM NaCl buffer of pH 6.0, referred to here as the native buffer.

### Far-UV CD measurements

Jasco spectropolarimeter (model J-1500) equipped with temperature controller (PTC-517) was used to measure far-UV CD spectra of proteins in the wavelength region 250–200 nm. CD spectra were obtained using a quartz cuvette (0.1 cm) at a scan rate of 100 nm/min with a response time of 1 s (1 nm bandwidth). The raw CD result was expressed in terms of mean residue ellipticity, [*θ*]_λ_ (deg cm^2^ dmol^−1^) using the following relationship,

1 $$\left[ \theta \right]_{\lambda } = M_{o} \theta_{\lambda } /10lc$$

where the *θ*_λ_ represents observed ellipticity in millidegrees, the mean residue weight of the protein is represented as *M*_o_, *c* is the protein concentration (mg/ml) and *l* is the path length (cm). Each spectrum of the protein was corrected for the corresponding blank solution.

### Analysis of denaturation curves

Data ([*θ*]_222_ versus *T*) obtained from thermal denaturation curves of a protein in 30 mM cacodylate buffer (pH 6.0) containing 100 mM NaCl (native buffer) were analyzed by reversible two-state mechanism, N (native) state ↔ D (denatured) state. Each denaturation curve was analyzed to obtain values of *T*_m_ (the temperature at which half of the protein is denatured) and Δ*H*_m_ (enthalpy change at *T*_m_) by a non-linear least-squares method according to the relation^25^,

2 $$y(T) = \frac{{y_{{\text{N}}} (T) + y_{{\text{D}}} (T)\exp [{{ - \Delta H_{{\text{m}}} } \mathord{\left/ {\vphantom {{ - \Delta H_{{\text{m}}} } {R({1 \mathord{\left/ {\vphantom {1 T}} \right. \kern-\nulldelimiterspace} T} - {1 \mathord{\left/ {\vphantom {1 {T_{{\text{m}}} )]}}} \right. \kern-\nulldelimiterspace} {T_{{\text{m}}} )]}}}}} \right. \kern-\nulldelimiterspace} {R({1 \mathord{\left/ {\vphantom {1 T}} \right. \kern-\nulldelimiterspace} T} - {1 \mathord{\left/ {\vphantom {1 {T_{{\text{m}}} )]}}} \right. \kern-\nulldelimiterspace} {T_{{\text{m}}} )]}}}}}}{{1 + \exp [{{ - \Delta H_{{\text{m}}} } \mathord{\left/ {\vphantom {{ - \Delta H_{{\text{m}}} } R}} \right. \kern-\nulldelimiterspace} R}({1 \mathord{\left/ {\vphantom {1 {T - {1 \mathord{\left/ {\vphantom {1 {T_{{\text{m}}} )]}}} \right. \kern-\nulldelimiterspace} {T_{{\text{m}}} )]}}}}} \right. \kern-\nulldelimiterspace} {T - {1 \mathord{\left/ {\vphantom {1 {T_{{\text{m}}} )]}}} \right. \kern-\nulldelimiterspace} {T_{{\text{m}}} )]}}}}}}$$

where *R* is the gas constant; *y*(*T*) is the observed optical property at a specific wavelength at temperature *T* K; and *y*_N_(*T*) and *y*_D_(*T*) denote the properties of N and D states of the protein at *T*, respectively. The temperature dependence of *y*_N_ and *y*_D_ are described by parabolic functions^26,27^,

2a $$y_{{\text{N}}} (T) = a_{{\text{N}}} + b_{{\text{N}}} T + c_{{\text{N}}} T^{2}$$

2b $$y_{{\text{D}}} (T) = a_{{\text{D}}} + b_{{\text{D}}} T + c_{{\text{D}}} T^{2}$$

where $a_{x} ,b_{{\text{x}}}$ and $c_{{\text{x}}}$ are temperature–independent coefficients of the protein (x = N for the native state and D for the denatured state of the protein), and *T* is the temperature in Kelvin. Using values of Δ*H*_m_, *T*_m_ and Δ*C*_p_, the Gibbs free energy change (Δ*G*_D_) at 25 °C (Δ*G*_D_^0^) was estimated with the help of Gibbs–Helmholtz equation,

3 $$\Delta G_{{\text{D}}}^{0} \left( T \right) = \Delta H_{{\text{m}}} \left( {\frac{{T_{{\text{m}}} {\text{ - }}298.2}}{{T_{{\text{m}}} }}} \right) - \Delta C_{{\text{p}}} \left[ {\left( {T_{{\text{m}}} - 298.2} \right) + T\ln \left( {\frac{298.2}{{T_{{\text{m}}} }}} \right)} \right]$$

## Results and discussion

### Characterization of L94G mutant of cyt *c* by SDS-PAGE and UV–Vis spectroscopy

**Figure 1:**
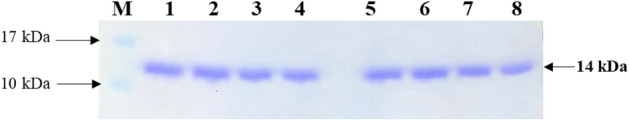
SDS-PAGE of the purified cyt *c*. Lanes M: Marker, 1–2: labeled WT cyt *c,* 3–4: labeled L94G mutant, 5–6: unlabeled WT cyt *c* and 7–8: unlabeled L94G mutant. Full gel image has been provided as Figure S1.

The purity of proteins was checked by measuring A_410_, the absorbance at 410 nm and A_280_, the absorbance at 280 nm. An absorbance (A_410_/A_280_) ratio > 4.0 is considered as pure protein preparation ^17^. We have observed that for all proteins this ratio was greater than 4.0. Figure 1 shows the SDS-PAGE of the unlabeled and labeled WT cyt *c* and its mutant L94G. It is seen in this figure that all four proteins gave a single band of ~ 12.5 kDa.

**Figure 2:**
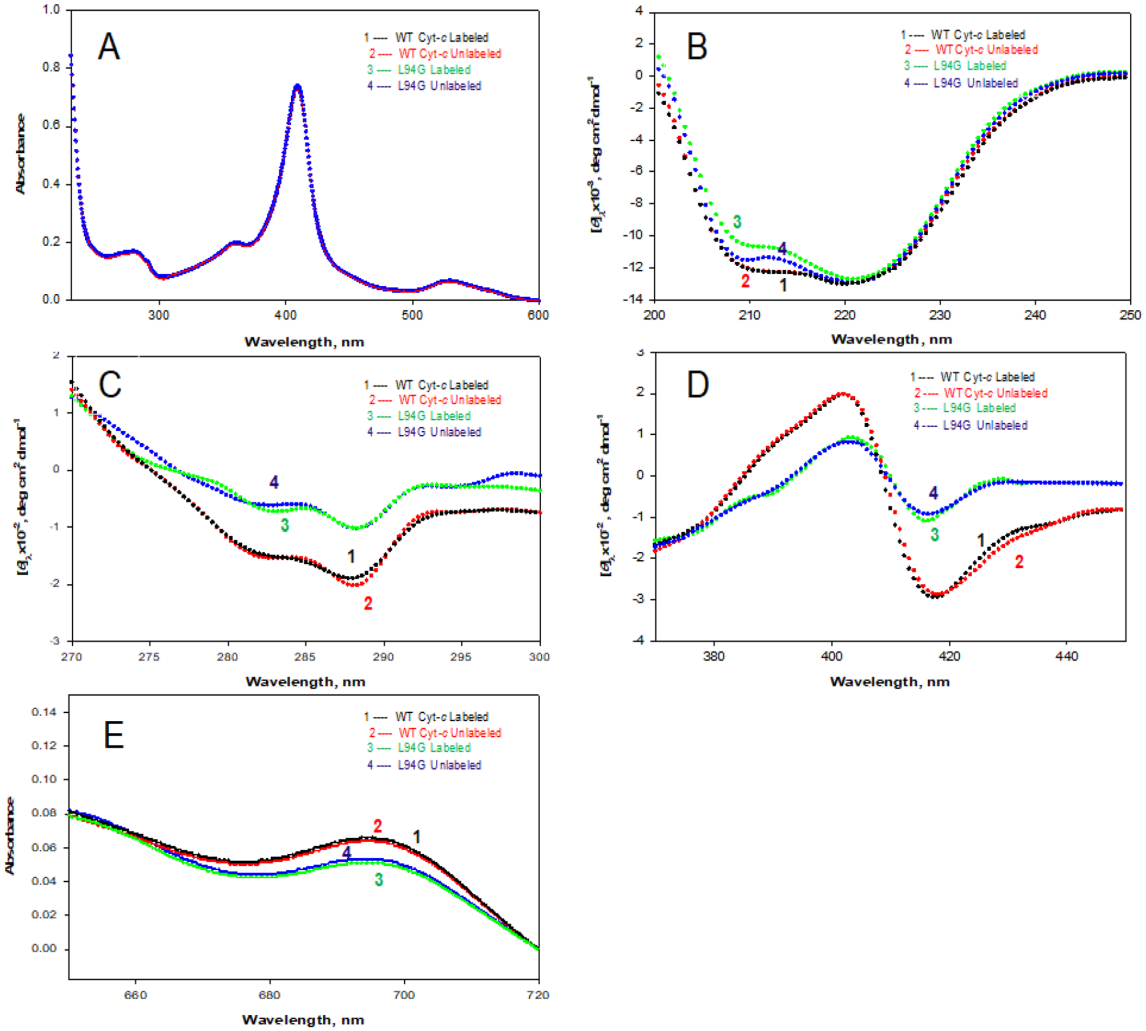
Spectroscopic characterization of WT cyt *c* and its L94G mutant. Protein concentration used for CD measurements was in the range of 14–18 μM. Protein concentration was in the range 6–8 μM and 75–80 μM for absorption measurements in the wavelength range of 600–230 nm and 720­–650 nm, respectively. **(A)** UV–Vis absorption spectra in the wavelength range 600–230 nm. **(B)** Far-UV CD Spectra in the wavelength range 250–200 nm. **(C)** Near-UV CD Spectra in the wavelength range 300–270 nm. **(D)** Soret CD Spectra in the wavelength range 450–370 nm. **(E)** Absorption spectra in the wavelength range 720–650 nm. All spectra were taken in the native buffer (30 mM cacodylate containing 100 mM NaCl, pH 6.0) at 25 ± 0.1 °C.

UV–Vis absorption spectra of the purified proteins were measured in the range 230–600 nm. The measured spectrum of the unlabeled WT cyt *c* was compared with the already published spectrum to determine the proper change in absorbance as the oxidation state of the protein changes. This comparison confirmed that the isolated red protein is, indeed, cyt *c*^28,29^. Figure 2A shows the UV–Vis spectra of the native oxidized WT cyt *c* (labeled and unlabeled) and its L94G mutant (labeled and unlabeled) displaying characteristic Soret band at 409 nm and a weaker Q band at 528 nm originated from the heme porphyrin^28,30^.

**Figure 3:**
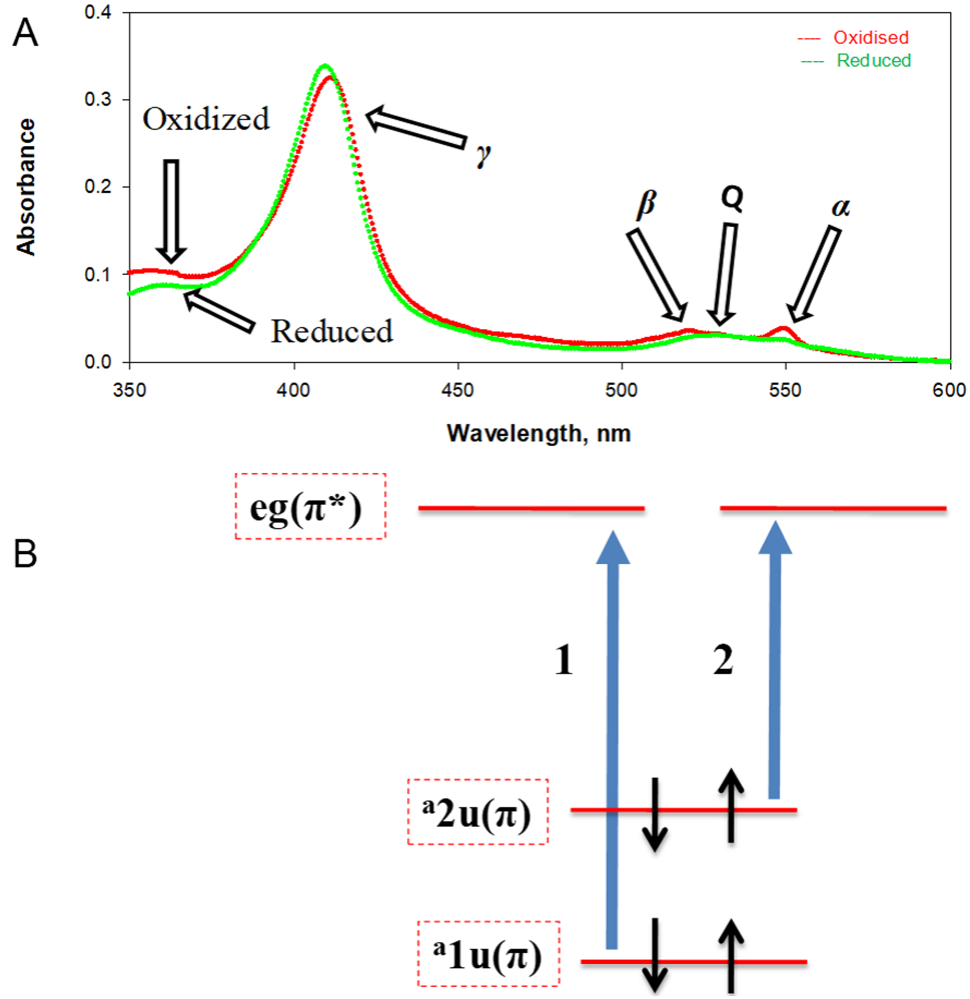
UV–Vis absorption spectra of the oxidized and reduced WT cyt *c* in the native buffer (30 mM cacodylate containing 100 mM NaCl (pH 6.0) at 25 ± 0.1 °C. **(A)** UV–Vis absorption spectra of the oxidized (red curve) and reduced (green curve) cyt *c*. **(B)** Molecular orbital diagram for the four-orbital model describing metalloporphyrin absorbances. The figure was redrawn from the ref.^34^.

The UV–Vis absorption spectra of both oxidized and reduced cyt *c* molecules are shown in Fig. 3A. These spectra are characteristics of the metalloporphyrin spectra. The more intense absorption band at 410 nm for the oxidized and 414 nm for the reduced protein is commonly referred to as the Soret band, while the less intense bands in the region of 500–560 nm are referred to as the α/β bands (Q band). These spectra can be explained by Gouterman’s four orbital models^31^. The four orbitals in this model are porphyrin π and π* orbitals. Figure 3B shows that the two highest occupied molecular orbitals have symmetry of ^a^u and ^a^2u (π orbitals) while the two lowest unoccupied molecular orbitals (π* orbitals) have symmetry of eg^32,33^. The α/β and Soret bands arise from the coupling of the transitions between π and π* orbitals (coupling of transitions 1 and 2 as shown in Fig. 3B). The α/β bands stem from one electronic transition but two separate vibrational transitions, explaining the two observed separate bands^34^.

### Circular dichroism spectral measurements

The far-UV CD spectrum provides information regarding the secondary structure (orientation of peptide backbone) of proteins. The far-UV CD spectrum of all α-proteins shows two negative minima at 208 nm and 222 nm and one positive maximum at 190 nm^35^. Far-UV CD spectra of both labeled and unlabeled WT cyt *c* and L94G mutant in the native buffer (30 mM cacodylate buffer (pH 6.0) containing 100 mM NaCl) were measured at 25 °C. In our case, due to high PM voltage, we could not obtain spectra of these proteins in the native buffer at wavelengths below 200 nm (Fig. 2B). Figure 2B shows far-UV CD spectra of the labeled (curve 1) and unlabeled (curve 2) of WT cyt *c* and labeled (curve 3) and unlabeled (curve 4) spectra of L94G mutant in the native buffer. Percent of α-helix content of WT and mutant proteins given in Table 1, were calculated using mean residue ellipticity ([*θ*]) values at 222 nm^36^ and 208 nm^37^.

**Table 1:** α-helical content of WT cyt *c* and its L94G mutant^a^.

Proteins	[*θ*]_222_, deg cm^2^ dmol^-1^	% α-helix^b^	[*θ*]_208_, deg cm^2^ dmol^-1^	% α-helix^c^
WT cyt *c* (unlabeled)	− 12,786 ± 280	39.4 ± 0.3	− 11,542 ± 145	38.2 ± 0.2
WT cyt *c* (labeled)	− 12,777 ± 246	39.4 ± 0.3	− 11,535 ± 136	38.1 ± 0.2
L94G (unlabeled)	− 12,804 ± 266	39.5 ± 0.3	− 11,161 ± 165	37.9 ± 0.2
L94G (labeled)	− 12,563 ± 320	39.2 ± 0.3	− 10,097 ± 180	37.8 ± 0.2

^a^± represents the error from the mean of errors of triplicate measurements.

^b^α-helical content estimated using equation given by Morrisett et al.^36^.

^c^α-helical content estimated using equation given by Greenfield & Fasman^37^.

It is interesting to note that (i) α-helical content of the unlabeled WT cyt *c* obtained from the CD measurements (Table 1) is excellent agreement with that determined by crystallographic studies^38^, and (ii) α-helical content of unlabeled and labeled WT protein is identical, suggesting that labeling does not affect the secondary structure of this protein. Far-UV CD spectra of both labeled and unlabeled L94G mutant were also analyzed for α-helical content (Table 1). This analysis led us to conclude that (i) α-helical content of the unlabeled mutant matches with the one previously reported^14^, and (ii) as observed in the case of the WT cyt *c*, labeling of the mutant does not cause any changes in the secondary structure.

Figure 2C (curve 1) shows the near-UV spectrum of the labeled WT cyt *c*, whereas (curve 2) shows unlabeled WT cyt *c*. Two minima at 282 and 289 nm of the WT protein are signatures of the natively folded cyt *c*^34,39^. Figure 2C also shows near-UV CD spectra of the labeled (curve 3) and unlabeled (curve 4) L94G mutant in the native buffer at 25 °C. It is seen in this figure that the magnitude of CD at 282 and 289 nm observed for the WT cyt *c* is significantly decreased on mutation (L94G). Near-UV CD spectra of the unlabeled WT cyt *c* and its unlabeled L94G mutant match with those previously reported^14^.

The near-UV CD of WT cyt *c* originates from tight packing of buried side chains of Trp, Phe and Tyr residues and two thioether bonds^39^. Interaction of Trp59 with one heme propionate gives rise to two negative peaks at 282 and 289 nm, and presence these negative cotton effects is a signature of the natively folded WT cyt *c*^39,40^. Labeling has no effect on the tertiary structure of proteins (WT cyt *c* and L94G mutant), for intensities of CD signals at 282 and 289 nm (characteristics of the native protein) of each labeled and unlabeled protein are unchanged (Fig. 2C). Spectra of L94G mutant labeled and unlabeled (curves 3 and 4 in Fig. 2C) lie above the spectrum of the native WT cyt *c* (Fig. 2C, curve 1 and curve 2). Thus, the near-UV CD measurements suggest a partial loss of tertiary structure of the WT cyt *c* on L94G mutation.

Effect of the mutation on the tertiary structure of WT cyt *c* was also monitored by measuring Soret CD spectra of the labeled and unlabeled proteins (WT cyt *c* and its L94G mutant) in the native buffer. Figure 2D shows Soret CD spectra of the labeled (curve 1) and unlabeled (curve 2) WT cyt *c* and those of labeled (curve 3) and unlabeled (curve 4) L94G mutant in the native buffer at 25 °C. The Soret region (390–450 nm) CD originates from non-covalent interactions between the heme-ring and polypeptide chain^38^. The presence of peaks at 405 and 416 nm in the CD spectrum of the native WT cyt *c* (Fig. 2D) is considered as fingerprint for the heme crevice structure in the native state^40,41^. CD positive band at 405 nm is a measure of heme-globin interaction and the spin state of heme iron^7,42^. Furthermore, a blue shift with an increase in intensity is observed due to changes in the heme-globin interaction and the spin state of Fe from low to high^43–45^. Met80-Fe and Phe82-heme interactions give rise to a negative band at 416 nm^46^. As it can be seen in Fig. 2D, labeled (curve 1) and unlabeled (curve 2) forms of WT cyt *c* show a positive peak at 405 nm which is slightly more intense than that of the labeled (curve 3) and unlabeled (curve 4) L94G mutant. This observation suggests that although L94G mutant maintains a low spin configuration similar to N state of WT cyt *c*, there is a slightly change in the spin state of the heme iron. Figure 2D also shows that intensity of the negative band centered at 416 nm of WT cyt *c* (curves 1and 2) is significantly decreased on mutation (curves 3 and 4), which suggests that Met80-Fe interaction is disrupted in L94G mutant and also the distance and orientation of Phe82 side chain, positioned on the Met80 side of heme plane, is perturbed*.* Measurements of CD spectra in the Soret region led to another conclusion that labeling of protein atoms does not affect the tertiary structure of the protein.

### Absorbance measurements

Figure 2E shows absorbance spectra of both WT cyt *c* and its L94G mutant under the native condition (pH 6.0 and 25 °C). Curves 1 and 2 represent absorbance spectra of the labeled and unlabeled WT cyt *c*, respectively, whereas curves 3 and 4 represent absorbance spectra of the labeled and unlabeled L94G mutant, respectively. Absorbance spectrum of WT cyt *c* in the wavelength range of 650–750 nm shows a broad single positive peak at 695 nm, which is a diagnostic test for the presence of Met80-Fe^+3^ axial bond in the protein^47^. Absorption characteristics of this bond is sensitive to distance between Met80 and heme Fe^48^. Figure 2E (curves 3 and 4) shows that there is a significant decrease in the intensity of the 695 nm absorbance band of the labeled and unlabeled L94G mutant with respect to the labeled and unlabeled WT cyt *c* (curves 1 and 2), which suggests a folded conformation with less-than-optimal Met80-Fe interaction. This observation is in agreement with that of the Soret CD spectrum at 416 nm (Fig. 2D).

Measurements of optical characteristics of L94G mutant in the native buffer by near-UV CD, Soret CD and visible absorbance spectroscopies led us to conclude that this mutant has partially perturbed tertiary structure in the vicinity of Trp59, Met80, Met82 and heme. Furthermore, from these studies, we also conclude that labeling does not cause any changes in the tertiary structures of proteins.

### Dynamic light scattering (DLS) measurements

To further characterize the native states of labeled and unlabeled L94G mutant with respect to the WT cyt *c* labeled and unlabeled, we carried out DLS measurements to determine the hydrodynamic radius (*R*_H_) and hydrodynamic volume, *V*_H_ (*V*_H_ = 4/3π*R*_H_^3^) of WT cyt *c* and its mutant L94G in the native buffer (pH 6.0) at 25 °C. Values of *R*_H_ and *V*_H_ obtained by DLS for the WT (labeled and unlabeled) cyt *c* and its labeled and unlabeled mutant are given in Table 2. It is seen in this table that each protein (labeled and unlabeled) has, within experimental errors, identical hydrodynamic properties. These observations suggest that labeling of atoms of proteins does not affect their gross conformations.

**Table 2:** Structural comparison of hydrodynamic properties between WT cyt *c* and its mutant L94G in the native buffer (pH 6.0) at 25°C^a^.

Proteins	*R*_H_, Å	*V*_H_, Å^3^
WT cyt *c* (unlabeled)	15.5 ± 0.3	15,604
WT cyt *c* (labeled)	15.3 ± 0.2	15,008
L94G (labeled)	17.4 ± 0.2	22,075
L94G (unlabeled)	17.2 ± 0.4	21,323

^a^± represents the error from the mean of errors of triplicate measurements.

### Thermal denaturation measurements of WT cyt *c* and L94G mutant

**Figure 4:**
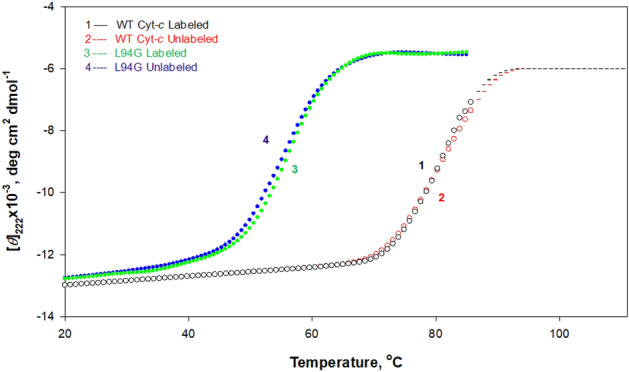
Thermal denaturation of WT cyt *c* and its mutant L94G at pH 6.0. Denaturation was monitored by [*θ*]_222_. Each dotted line is drawn with best fitted parameters according to Eq. (2).

To compare the thermodynamic stability of WT cyt *c* (labeled and unlabeled) with that of L94G mutant (labeled and unlabeled), thermal denaturation of both the proteins in the native buffer was monitored by following changes in [*θ*]_222_ in the temperature range 20–85 °C. Figure 4 shows these thermal denaturation curves. It is seen in this figure that denaturation of both labeled and unlabeled WT cyt *c* is not complete in the temperature range of measurements. To check the reversibility of the thermal denaturation of both proteins, spectra at 25 °C were taken before and after heating.

**Figure 5:**
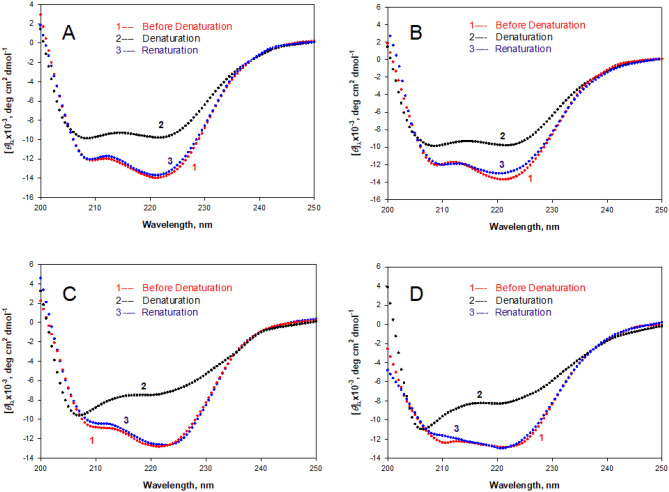
Reversibility of heat-induced denaturation of the labeled and unlabeled WT cyt *c* and its mutant L94G in the native buffer. Panels **A** (unlabeled) and **B** (labeled) show spectra of the unheated native (curve 1), denatured (curve 2) and renatured (curve 3) WT cyt *c*. Panels **C** (unlabeled) and **D** (labeled) show spectra of the unheated native (curve 1), denatured (curve 2) and renatured (curve 3) L94G mutant.

Figures 5A,B show denaturation and renaturation curves of the unlabeled and labeled WT cyt *c*. It is seen here that protein denatured at 85 °C regains its native far-UV CD characteristics on cooling to 25 °C. It has been shown earlier that heat-induced denaturation of the unlabeled WT cyt *c* is reversible^14,49^. To check the reversibility of heat-induced denaturation of the labeled L94G mutant and that of the unlabeled L94G mutant, each solution was heated to 65 °C at which protein is completely denatured, followed by cooling the solution to 25 °C. Figures 5C,D show that the CD spectrum of the renatured protein is identical to that of the unheated protein solution suggesting that thermal denaturation of the labeled and unlabeled mutant is reversible.

It has been shown earlier ^14,50^ that heat-induced denaturation of unlabeled proteins (WT cyt *c* and L94G mutant) is reversible and follows a two-state mechanism. Heat-induced denaturation curves of WT cyt *c* and L94G mutant (Fig. 4) were analyzed for thermodynamic parameters, *T*_m_ (temperature at the midpoint of denaturation) and Δ*H*_m_ (enthalpy change at *T*_m_), using Eq. (2). These values of thermodynamic parameters for all proteins are given in Table 3. In this table, reported calorimetric *T*_m_ and Δ*H*_m_ values of the unlabeled proteins (WT cyt *c* and L94G mutant) are also given in parenthesis^14,50^. It is seen in this table that values of these thermodynamic parameters of the unlabeled WT and mutant proteins are in excellent agreement with those obtained from DSC (differential scanning calorimetry) measurements reported earlier^14,50^. It should be noted that the heat-induced denaturation curves of labeled and unlabeled WT cyt *c* are not complete in the measurable temperature range. However, we have analyzed these transition curves according to Eq. (2). As seen in Table 3, since thermodynamic parameters of each unlabeled and labeled protein are in excellent agreement with those reported earlie r^14,50^, our analysis of denaturation curves of labeled proteins (WT cyt *c* and L94G mutant) assuming two-state mechanism, is valid.

**Table 3:** Thermodynamic parameters associated with the heat-induced denaturation of WT cyt *c* and its L94G mutant in the native buffer (pH 6.0).^a,b^.

Proteins	Thermodynamic parameters			
	*T*_m_, °C	∆*H*_m_, kcal mol^−1^	∆*C*_p_^c^, kcal mol^-1^ K^−1^	∆*G*_D_^0^, kcal mol^−1^
WT cyt *c* (unlabeled)	81.80 ± 0.40 (80.40 ± 0.20)	98 ± 2 (97 ± 2)	1.22 ± 0.07 (1.22 ± 0.07)	9.81 ± 0.18 (9.48 ± 0.19)
WT cyt *c* (labeled)	82.50 ± 0.34	98 ± 2	1.22 ± 0.07	9.84 ± 0.15
L94G mutant (unlabeled)	55.00 ± 0.20 (54.10 ± 0.30)	72 ± 3 (72 ± 2)	0.70 ± 0.2 (0.70 ± 0.2)	5.56 ± 0.14 (5.6 ± 0.2)
L94Gmutant (labeled)	54.40 ± 0.10	72 ± 3	0.70 ± 0.2	5.48 ± 0.13

^a^± represents the deviation from the mean value of triplicate measurements.

^b^In the presence of 30 mM sodium cacodylate and 100 mM NaCl (pH 6.0).

^c^Values taken from^50,51^. Values in parenthesis are from DSC measurements.

The Gibbs free energy change at 25 °C (Δ*G*_D_^0^) was estimated using values of *T*_m_, Δ*H*_m_ and Δ*C*_p_ in the Gibbs–Helmholtz Eq. (3). It should be noted that in the estimation of Δ*G*_D_^0^, we have used values of 1.22 kcal mol^-1^ K^-1^ for Δ*C*_p_ of WT cyt *c*^51^, and 0.7 kcal mol^-1^ K^-1^ for Δ*C*_p_ of L94G mutant^14,50^. The values of Δ*G*_D_^o^ of all proteins thus obtained are given in Table 3. Thus, measurements of the effect of the mutation on stability of WT cyt *c* in terms of *T*_m_ and Δ*G*_D_^0^ revealed that unlabeled L94G mutant has *T*_m_ and Δ*G*_D_^0^ values which are respectively 26.8 ± 0.2 °C and 4.25 ± 0.04 kcal mol^-1^ less than those of WT cyt *c*. The labeled L94G mutant has *T*_m_ and Δ*G*_D_^0^ values which are respectively 28.1 ± 0.24 °C and 4.36 ± 0.02 kcal mol^-1^ less than those of WT cyt *c* (see Table 3).

It is interesting to recall a study which reported the results of calorimetric measurements of ∆*H*_m_ of several proteins in H_2_O and D_2_O^16^. From this study of solvent isotope effect on protein stability, it was concluded that ∆*H*_m_ of proteins in the presence of D_2_O significantly decreased. Based on model compound data, it was argued that a decrease in ∆*H*_m_ is due to the difference in strength of hydrogen bonding interactions between D_2_O molecules and between normal water molecules. Cyt *c* has about 68 hydrogen bonds in which N atoms are involved in hydrogen bond formation^38^. Results shown in Tables 1,2and3 suggest that labeling of N atoms (^15^N) of WT cyt *c* and its mutant does not affect the secondary structure, hydrodynamic volume and thermodynamic parameters (∆*H*_m_, *T*_m_ and ∆*G*_D_^0^) of proteins, respectively. Thus, contrary to proteins in D_2_O, labeling of N atoms does not affect the strength of hydrogen bonds in WT cyt *c* and its mutant, for ∆*H*_m_ of proteins is unperturbed.

## Conclusions

The above discussion on the structural characteristics of WT cyt *c* (labeled and unlabeled) and those of its mutant L94G (labeled and unlabeled) shows that mutant has (i) α-helical content which is almost identical to that present in the WT protein, (ii) partially perturbed tertiary structure, (iii) newly exposed hydrophobic patches, and (iv) hydrodynamic radius which is 11% larger than that of the WT protein. These observations suggest that mutant L94G in the native buffer exhibits all features similar to those of the molten globule state^52–54^. Measurements of thermodynamic parameters show that L94G mutant is less stable than the WT protein. From these studies, we conclude that labeling does not cause any changes in the secondary and tertiary structures and thermodynamic stability of proteins.

## Supplementary Information

Supplementary Information

## References

[1] Levinthal C (1968). Are there pathways for protein folding?. J. Chem. Phys..

[2] Kim PS, Baldwin RL (1982). Specific intermediates in the folding reactions of small proteins and the mechanism of protein folding. Annu. Rev. Biochem..

[3] Kim PS, Baldwin RL (1990). Intermediates in the folding reactions of small proteins. Annu. Rev. Biochem..

[4] Naiyer A, Hassan MI, Islam A, Sundd M, Ahmad F (2015). Structural characterization of MG and pre-MG states of proteins by MD simulations, NMR, and other techniques. J. Biomol. Struct. Dyn..

[5] Arai M, Kuwajima K (2000). Role of the molten globule state in protein folding. Adv. Protein Chem..

[6] Christensen H, Pain RH (1991). Molten globule intermediates and protein folding. Eur. Biophys. J..

[7] Ubaid-Ullah S (2014). Effect of sequential deletion of extra N-terminal residues on the structure and stability of yeast iso-1-cytochrome-c. J. Biomol. Struct. Dyn..

[8] Zaidi S (2017). Denatured states of yeast cytochrome c induced by heat and guanidinium chloride are structurally and thermodynamically different. J. Biomol. Struct. Dyn..

[9] Zaidi S, Hassan MI, Islam A, Ahmad F (2014). The role of key residues in structure, function, and stability of cytochrome-c. Cell Mol. Life Sci..

[10] Khan MKA (2009). A single mutation induces molten globule formation and a drastic destabilization of wild-type cytochrome c at pH 6.0. JBIC.

[11] Rahaman H (2015). Heterogeneity of equilibrium molten globule state of cytochrome c induced by weak salt denaturants under physiological condition. PLoS ONE.

[12] Khan MKA (2010). Conformational and thermodynamic characterization of the premolten globule state occurring during unfolding of the molten globule state of cytochrome c. J. Biol. Inorg. Chem..

[13] Rahaman H (2008). Sequence and stability of the goat cytochrome c. Biophys. Chem..

[14] Alam Khan MK (2009). A single mutation induces molten globule formation and a drastic destabilization of wild-type cytochrome c at pH 6.0. J. Biol. Inorg. Chem..

[15] Scheiner S, Čuma M (1996). Relative stability of hydrogen and deuterium bonds. J. Am. Chem. Soc..

[16] Naiyer A, Islam A, Hassan MI, Ahmad F, Sundd M (2019). Backbone and side chain (1)H, (15)N and (13)C chemical shift assignments of the molten globule state of L94G mutant of horse cytochrome-c. Biomol. NMR Assign.

[17] Patel CN, Lind MC, Pielak GJ (2001). Charaterization of horse cytochrome *c* expressed in Escherichia coli. Protein Expr. Purif..

[18] Naiyer A (2020). Heme-iron ligand (M80-Fe) in cytochrome c is destabilizing: combined in vitro and in silico approaches to monitor changes in structure, stability and dynamics of the protein on mutation. J. Biomol. Struct. Dyn..

[19] Laemmli UK (1970). Cleavage of structural proteins during the assembly of the head of bacteriophage T4. Nature.

[20] McPhie P (1972). Dialysis. Methods Enzymol..

[21] Bedard S, Krishna MM, Mayne L, Englander SW (2008). Protein folding: independent unrelated pathways or predetermined pathway with optional errors. Proc. Natl. Acad. Sci. U S A.

[22] Goto Y, Takahashi N, Fink AL (1990). Mechanism of acid-induced folding of proteins. Biochemistry.

[23] Butt WD, Keilin D (1962). Absorption spectra and some other properties of cytochrome c and of its compounds with ligands. Proc. R Soc. Lond. Ser. B Biol. Sci..

[24] Margoliash E, Frohwirt N (1959). Spectrum of horse-heart cytochrome c. Biochem. J..

[25] Santoro MM, Bolen DW (1988). Unfolding free energy changes determined by the linear extrapolation method. 1. Unfolding of phenylmethanesulfonyl alpha-chymotrypsin using different denaturants. Biochemistry.

[26] Sinha A, Yadav S, Ahmad R, Ahmad F (2000). A possible origin of differences between calorimetric and equilibrium estimates of stability parameters of proteins. Biochem. J..

[27] Yadav S, Ahmad F (2000). A new method for the determination of stability parameters of proteins from their heat-induced denaturation curves. Anal. Biochem..

[28] Dixon M, Hill R, Keilin D (1931). The absorption spectrum of the component c of cytochrome. Proc. R. Soc Lond. Ser. B Contain. Papers Biol. Char..

[29] Dunbar J, Yennawar HP, Banerjee S, Luo J, Farber GK (1997). The effect of denaturants on protein structure. Protein Sci..

[30] Oellerich S, Wackerbarth H, Hildebrandt P (2002). Spectroscopic characterization of nonnative conformational states of cytochrome c. J. Phys. Chem. B.

[31] Gouterman M (1978). The porphyrins.

[32] Dilung II, Kapinus EI (1978). The photonics of porphyrin molecules. Russ. Chem. Rev..

[33] Suslick KS, Watson RA (1992). The photochemistry of chromium, manganese and iron pophyrin complexes. New J. Chem..

[34] Moore, G. R., Pettigrew, G. W. . *Cytochrome c: Evolution, Structural and Physiological Aspects*. (1990).

[35] Holzwarth G, Doty P (1965). The ultraviolet circular dichroism of polypeptides. J. Am. Chem. Soc..

[36] Morrisett JD, David JS, Pownall HJ, Gotto AM (1973). Interaction of an apolipoprotein (apoLP-alanine) with phosphatidylcholine. Biochemistry.

[37] Greenfield N, Fasman GD (1969). Computed circular dichroism spectra for the evaluation of protein conformation. Biochemistry.

[38] Bushnell GW, Louie GV, Brayer GD (1990). High-resolution three-dimensional structure of horse heart cytochrome c. J. Mol. Biol..

[39] Davies AM (1993). Redesign of the interior hydrophilic region of mitochondrial cytochrome c by site-directed mutagenesis. Biochemistry.

[40] Myer, Y. P. Conformation of cytochromes. 3. Effect of urea, temperature, extrinsic ligands, and pH variation on the conformation of horse heart ferricytochrome c. *Biochemistry* **7**, 765–776 (1968). 10.1021/bi00842a035 5689422

[41] Oellerich S, Wackerbarth H, Hildebrandt P (2002). Spectroscopic characterization of nonnative conformational states of cytochrome c. J. Phys. Chem. B.

[42] Dyson HJ, Beattie JK (1982). Spin state and unfolding equilibria of ferricytochrome c in acidic solutions. J. Biol. Chem..

[43] Fedurco, M. *et al.* The heme iron coordination of unfolded ferric and ferrous cytochrome c in neutral and acidic urea solutions. Spectroscopic and electrochemical studies. *Biochim Biophys Acta* **1703**, 31–41, 10.1016/j.bbapap.2004.09.013 (2004). 10.1016/j.bbapap.2004.09.013 15588700

[44] Myer, Y. P., MacDonald, L. H., Verma, B. C. & Pande, A. Urea denaturation of horse heart ferricytochrome c. Equilibrium studies and characterization of intermediate forms. *Biochemistry* **19**, 199–207 (1980). 10.1021/bi00542a030 6243472

[45] Sanghera N, Pinheiro TJ (2000). Unfolding and refolding of cytochrome c driven by the interaction with lipid micelles. Protein Sci..

[46] Pielak GJ, Mauk AG, Smith M (1985). Site-directed mutagenesis of cytochrome c shows that an invariant Phe is not essential for function. Nature.

[47] Schejter, A. & George, P. The 695-nm. Band of Ferricytochrome C and Its Relationship to Protein Conformation. *Biochemistry* **3**, 1045–1049 (1964). 10.1021/bi00896a006 14220663

[48] Schejter A (2006). The redox couple of the cytochrome c cyanide complex: the contribution of heme iron ligation to the structural stability, chemical reactivity, and physiological behavior of horse cytochrome c. Protein Sci..

[49] Privalov PL (1979). Stability of proteins: small globular proteins. Adv. Protein Chem..

[50] Alam Khan, M. K. *et al.* Conformational and thermodynamic characterization of the premolten globule state occurring during unfolding of the molten globule state of cytochrome c. *J. Biol. Inorg. Chem.* **15**, 1319–1329, 10.1007/s00775-010-0691-5 (2010). 10.1007/s00775-010-0691-5 20694825

[51] Khan SH (2017). Effect of conservative mutations (L94V and L94I) on the structure and stability of horse cytochrome c. Arch. Biochem. Biophys..

[52] Kuwajima K (1989). The molten globule state as a clue for understanding the folding and cooperativity of globular-protein structure. Proteins.

[53] Naiyer, A., Hassan, M. I., Islam, A., Sundd, M. & Ahmad, F. Structural characterization of MG and pre-MG states of proteins by MD simulations, NMR, and other techniques. *J. Biomol. Struct. Dyn.*10.1080/07391102.2014.999354 (2015). 10.1080/07391102.2014.999354 25586676

[54] Qureshi SH, Moza B, Yadav S, Ahmad F (2003). Conformational and thermodynamic characterization of the molten globule state occurring during unfolding of cytochromes-c by weak salt denaturants. Biochemistry.

