# Supplementary material for: Stability of uniformly labeled (13C and 15N) cytochrome c and its L94G mutant
Source: Sci Rep. 2021 Mar 24;11:6804. doi: 10.1038/s41598-021-86332-w (PMC7990917; doi:10.1038/s41598-021-86332-w)
Supplement: Supplementary file 1 — Supplementary Information [file 41598_2021_86332_MOESM1_ESM.docx]

Supplementary File

**Stability of uniformly labeled (^13^C and ^15^N) cytochrome *c* and its L94G mutant**

**Abdullah Naiyer^1^, Bushra Khan^1^, Afzal Hussain^2^, Asimul Islam^1^, Mohamed F. Alajmi^2^, Md. Imtaiyaz Hassan^1^,** **Monica Sundd^3,*^, and Faizan Ahmad^1^,***

^1^ Centre for Interdisciplinary Research in Basic Sciences, Jamia Millia Islamia, Jamia Nagar, New Delhi 110025, India.

^2^ Department of Pharmacognosy College of Pharmacy, King Saud University, Riyadh 11451 KSA.

^3^NMR-II Lab, National Institute of Immunology, Aruna Asaf Ali Marg, New Delhi –110067, India

***** Correspondence: monicasundd@nii.res.in (M. S.); [fahmad@jmi.ac.in](mailto:fahmad@jmi.ac.in) (F. A.)


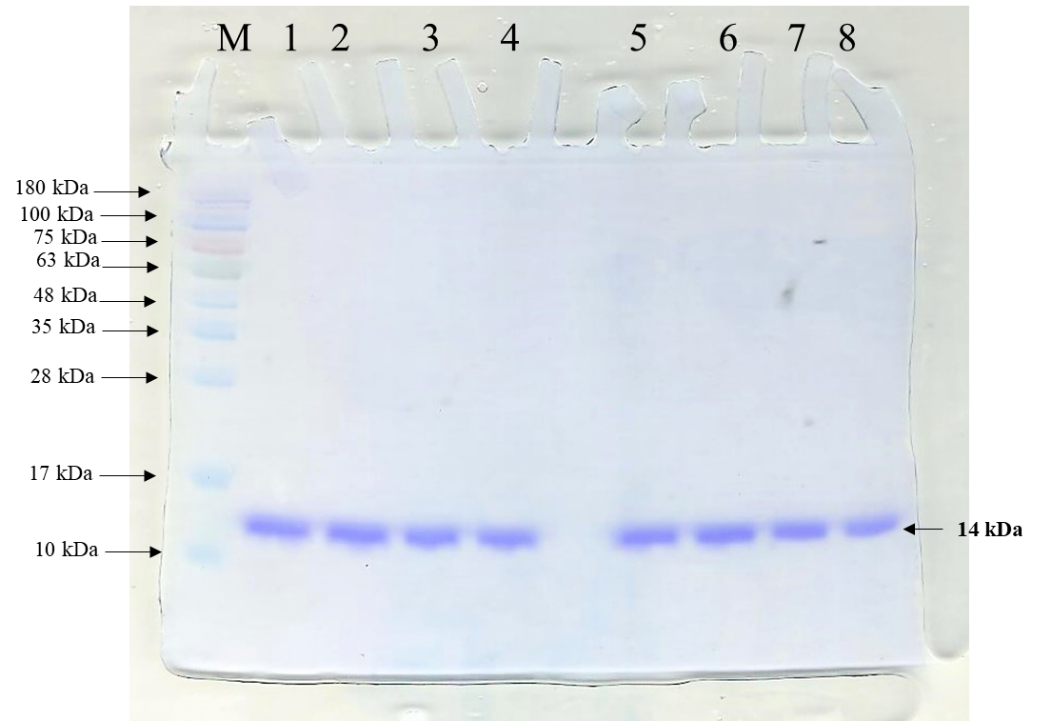


**Figure S1:** SDS-PAGE of the purified cyt *c*. Lanes M: Marker, 1-2: labeled WT cyt *c,* 3-4: labeled L94G mutant, 5-6: unlabeled WT cyt *c* and 7-8: unlabeled L94G mutant.
